# SARS-CoV-2 spike peptide analysis reveals a highly conserved region that elicits potentially pathogenic autoantibodies: implications to pan-coronavirus vaccine development

**DOI:** 10.3389/fimmu.2025.1488388

**Published:** 2025-02-25

**Authors:** Marilyn Diaz, Zbignew Mikulski, Dan Leaman, Angel Gandarilla, Nathalia Da Silva, Annie Verkoczy, Jinsong Zhang, Laurent Verkoczy

**Affiliations:** ^1^ Department of Vaccine Research and Development, Applied Biomedical Science Institute, San Diego, CA, United States; ^2^ Microscopy and Histology Core Facility, La Jolla Institute for Immunology, La Jolla, CA, United States; ^3^ Department of Immunology and Microbiology, The Scripps Research Institute, La Jolla, CA, United States

**Keywords:** COVID-19 infection, vaccine, B cells, immune, autoantibodies, autoimmunity

## Abstract

The SARS-CoV-2 pandemic, while subsiding, continues to plague the world as new variants emerge. Millions have died, and millions more battle with the debilitating symptoms of a clinical entity known as long Covid. The biggest challenge remains combating an ever-changing variant landscape that threatens immune evasion from vaccine and prior infection-generated immunity. In addition, the sequelae of symptoms associated with long Covid almost certainly point to multiple pathologies that range from direct damage to organs during infection to a potential role for infection-induced autoreactive antibodies in promoting autoimmune-like conditions in these patients. In this study, a peptide scan of the SARS-CoV-2 spike protein was done to detect novel, highly conserved linear epitopes that do not elicit autoantibodies. We identified eight predicted linear epitopes capable of eliciting anti-spike IgG antibodies. Immunizations alternating peptide conjugated to KLH with the full trimer yielded the highest antibody levels, but homologous immunization with some of the peptides also yielded high levels when an additional immunization step was added. Of all regions tested, the stem helix adjacent to the heptad repeat 2 (HR2) region also elicited high levels of autoreactive antibodies to known autoantigens in common systemic autoimmune disorders such as lupus and scleroderma and may contribute to the long Covid syndrome seen in some patients. Implications to vaccine design are discussed.

## Introduction

Although a respiratory virus, SARS-CoV-2 infections impact multiple organs, particularly among those severely afflicted. Cardiovascular, renal, and neurological complications are not uncommon with COVID-19, with many leading to long Covid (1). Given the multiple ways SARS-CoV-2 can cause significant organ damage in some people, and the fact that new variants continue to emerge in parts of the world with unmitigated infection, the development of new vaccines that can protect from existing and future new variants is needed. An attractive approach is to develop safe pan-coronavirus vaccines that target regions that are highly conserved across strains due to functional or structural constraints.

A potential impediment to pan-coronavirus vaccine efforts is that some of the highly conserved regions in other viruses, notably HIV-1, are associated with the generation of autoantibodies or B-cell development blockade from tolerance checkpoints that lead to immune repertoire gaps, rendering those regions difficult to target (2–4). To identify potential highly conserved SARS-CoV-2 targets that are immunogenic and capable of inducing anti-spike IgG antibodies, but do not elicit autoreactive antibodies, we scanned the entire spike protein amino acid sequence for linear epitopes that meet these requirements. We found that multiple linear epitopes elicited anti-spike antibodies, but one region also elicited particularly high levels of potentially pathogenic autoantibodies. These results will help inform viable target regions for developing a safe potential pan-coronavirus vaccine.

## Materials and Methods

### Peptide analysis and design

A peptide scan of the spike region sequence (UniProt: P0DTC2 · SPIKE_SARS2) for potential linear B-cell epitopes was performed using the BepiPred Linear Epitope Prediction 2.0 program (5). Candidate epitopes were compared for homology against SARS-1 using the T-Coffee (6) web interface to identify highly conserved potential epitopes. Additional candidates were also considered based on previous data on SARS-1 as potentially eliciting autoantibodies or that may promote antibody-dependent enhancement (7) or that fell in regions predicted to be exposed as linear epitopes based on protein secondary and tertiary structure analysis.

### Immunogen preparation

SARS-CoV-2 B epitope peptide synthesis was done by GenScript (Piscataway, NJ) to 95% purity after conjugation to KLH on cysteine. Trifluoroacetic acid was removed in all peptide preparations and AccuPep qualitative solubility test was performed. SARS-CoV2 Hexapro spike proteins (containing six prolines to stabilize the trimer in pre-fusion state) (8) were transiently expressed in ExpiCHO cells (Thermo Fisher Scientific). Briefly, cells were grown to a density of 6 × 10^6^/mL in 25 mL of ExpiCHO Expression Medium at 37°C, with 135 rpm shaking and 8% CO_2_. The ExpiFectamine CHO transfection reagent and 25 μg of plasmid DNA were complexed according to the manufacturer’s instructions and added to the cells. After 24 h, ExpiCHO Feed and Enhancer were added and cells were cultured for an additional 6 days at 32°C, with 120 rpm shaking and 5% CO_2_. Culture supernatants were harvested, cleared by centrifugation, and 0.45-μm filtered. Spike proteins were purified from the culture supernatants using GNL Agarose (Vector Labs) and eluted using 1 M methyl α-D-mannopyranoside (Sigma). Proteins were then buffer exchanged into PBS using 100 kDa MWCO Amicon concentrators (Millipore) and further purified by size exclusion chromatography using a Superdex 200 Increase 10/300 GL column (Cytiva). Prior to immunizations, 25 μg of purified peptide-KLH or SARS-CoV-2 Hexapro spike proteins was formulated in Alum (1:3 ratio, Imject Alum, Cat# 77161, Thermo Fisher Scientific, Waltham, MA) plus TLR agonists 20 μg of CpG (ODN 1826 VacciGrade, Cat# vac-1826-1, Invivogen, San Diego, CA) and 10 μg of MPLA (MPLA-SM VacciGrade, Cat# vac-mpla, Invivogen) in 200-μL volumes, and after continuous mixing for 30 min, Imject Alum was added.

### Immunizations

Eight- to 10-week-old BALB/c mice (distributed equally among genders) were purchased from Jackson Laboratories and allowed to acclimate to our facilities for at least 10 days prior to immunizations. Groups of mice were primed and boosted following one of the following regimens: (1) 25 µg of specific peptide, (2) part of the S1 subunit of SARS-CoV-2 (aa 15 through 769; involved in receptor mediated binding) (Trenzyme SKU:P2020-022), (3) the entire S2 subunit (aa 770 through 1376) twice (R&D Systems, Cat# 10584-CV), or (4) the SARS-CoV-2 spike protein (Trenzyme SKU:P2020-029). In parallel, another series of experiments used mice that were primed with the Hexapro trimer then boosted with one of the specific peptides above. A second boost was done for all mice (except those immunized with trimer or subunits alone) using the corresponding peptide and serum collected 10 days later (P3; see **Supplementary Figure S1** for all immunization schemes). The first boost was done after 16 days post prime whereas a subsequent boost was done at least 3 months later as depicted in **Supplementary Figure S1**. Pre-bleeds were collected from all mice prior to the prime, and each post-bleed was collected 10 days after each immunization. All mice used in this study were housed at the ABS Animal Facility in a specific pathogen-free environment with 12-h light/dark cycles at 20–25°C following American Association of Laboratory Animal Care guidelines. For all immunization groups, a minimum of four mice per group were used, and single-site injections were administered intraperitoneally.

### ELISA for anti-spike or anti-peptide binding antibodies

Enzyme-linked immunosorbent assay (ELISA) measurements of antibodies binding to SARS-CoV-2 peptide epitopes in serum were determined using high-binding microtiter plates coated with PBST (PBS with 0.05% Tween 20) and 2 μg/mL SARS-CoV-2 peptide-BSA proteins (same peptides used in immunization but conjugated to BSA instead of KLH) (GenScript). Anti-SARS-CoV-2 spike Ab titers were determined in plates coated with PBST and 2 μg/mL of full-length SARS-CoV-2 spike trimer. After washing twice with PBST, plates were blocked with 1% BSA in PBST for 2 h at room temperature, and again washed twice with PBST, after which serum serial dilutions were applied to each well and incubated overnight at 4°C. The plates were then washed four times with PBST, and AP-conjugated anti-mouse Kappa or IgG-specific reagents were added to each well and again incubated overnight at 4°C. After washing four times with PBST, the signals were detected by the AttoPhos^®^ AP Fluorescent Substrate System (Promega, Madison, WI) according to the manufacturer’s manual.

### Antinuclear antibodies test

Antinuclear antibodies (ANA) tests were performed using KALLESTAD HEp-2 Cell Line Substrate slides (Bio-Rad, Cat# 26103). Mouse serum samples were thawed on ice, centrifuged at 15,000 *g* for 15 min at 4°C, and diluted at 1:100 in PBS. Samples were arrayed in 96 polypropylene well plates, and 40 µL of diluted serum was applied to wells using a multichannel pipettor. ANA slides were incubated in a humidified chamber at room temperature for 30 min. Each well was gently rinsed with a stream of PBS and slides were washed for an additional 10 min in a Coplin jar filled with PBS. After blotting the excess PBS from around the wells with blotting strips, samples were stained with 40 µL of goat anti-mouse IgG Alexa Fluor 488 secondary antibodies (1:500 in PBS, Thermo Fisher Scientific Cat# A-11001, RRID: AB_2534069) and Hoechst 33342 (1:2000, Thermo Fisher Scientific Cat# H3570) for 30 min in a humidified chamber at room temperature. Samples were washed as described and embedded with ProLong Gold mounting medium (Thermo Fisher Scientific Cat# P36930) and #1.5 24×60 mm coverslips. Serum samples from autoimmune MRL/MpJ-Faslpr/J RRID: IMSR_JAX:000485 were used as positive controls and naive C57BL/6J mice were used as negative controls. Additionally, pre-bleed serum from all experimental mice (obtained before their immunization) was tested as baseline controls. Samples were imaged on a ZEISS AxioScan.Z1 slide scanner equipped with 10× 0.45 NA or 20× 0.8 NA dry objective, a Colibri 7 light source, and an Orca Flash4.0 V2 digital camera. Images were analyzed in ZEN 3.6 (Zeiss) and QuPath (9). Patterns of staining were compared to images presented on ANApatterns.org, the official website for the International Consensus on ANA Patterns (ICAP) (10).

### Autoantibody detection array

The RayBiotech human autoimmune disease IgG autoantibody detection array G1 (Cat# PAF-AIDG-G1) was used according to the manufacturer’s protocol. To detect binding of mouse immunoglobulins to spotted autoimmunogens, the biotinylated anti-human IgG antibody was substituted with Biotin-SP AffiniPure Goat Anti-Mouse IgG, Fcγ fragment specific (1:1,000, Jackson ImmunoResearch Labs Cat# 115-065-071, RRID: AB_2338564). The slide was sent to RayBiotech for data collection and quantification. Intensity counts from individual autoimmunogen spots were measured in triplicate and normalized to internal positive control spots of Biotin-BSA. Negative control spots included water and 0.1% BSA in PBS. Data analysis was performed in Excel.

## Results

### Spike region analysis and peptide design

Our multipronged analysis of the spike region resulted in the identification of 14 peptide candidates that were predicted to be at least partially exposed as linear epitopes and, in some cases, highly conserved across all identified SARS-CoV-2 variants of importance or with SARS-1 (**Supplementary Figure S2**). To test their immunogenicity, we designed a two-arm study in which we conjugated all peptides to KLH and adjuvanted them as described above (combination of Alum and TLR agonists). In one arm, heterologous immunizations were done wherein the peptides were individually administered as a boost 30 days after priming with either the full trimer stabilized with six prolines or the prototype spike protein used in vaccines (with two extra prolines) obtained from Trenzyme (11, 12). On the other arm, homologous immunizations were done wherein mice were both primed and boosted either using the same individual peptide or the same protein subunit as the prime (**Supplementary Figure S1**).

### Generation of anti-peptide and anti-spike IgG antibodies following immunizations

**Figure 1:**
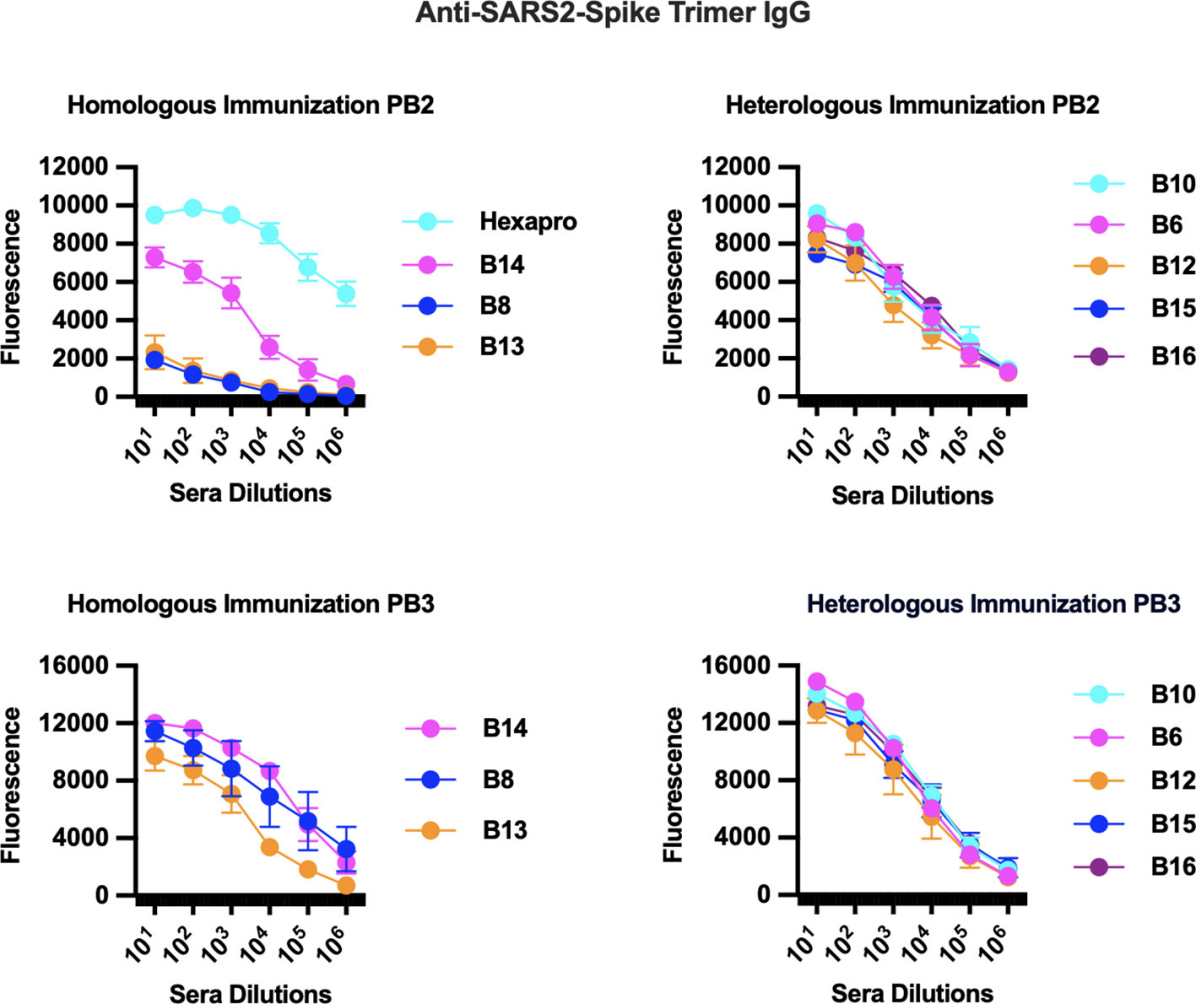
Immunization studies with predicted linear peptides reveal that some peptides elicited anti-spike IgG antibodies. Homologous [peptide or trimer (Hexapro) only] and heterologous (primed with trimer then boosted with specific peptide) immunizations (**Supplementary Figure S1**) were examined by ELISA for eliciting anti-spike IgG antibodies. Peptides that elicited anti-spike IgG were B10, B15, and B16 (near the receptor binding domain), B12 (near the fusion peptide domain), B14 (novel S1 region), B8 and B13 (both in the stem helix region leading to the HR2), and B6 (also in S2). Homologous immunizations generated robust anti-spike IgG titers after two boosts (P3) while heterologous immunizations that included the trimer as a primer generated the highest levels. Homologous immunization with the full trimer (Hexapro) generated the highest anti-spike IgG levels after a single boost.

**Figure 2:**
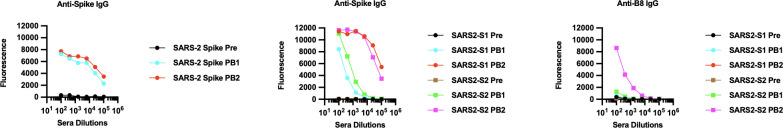
Anti-spike IgG ELISA of pooled sera collected from immunizations with either the full trimer or with spike subunits and tested by ELISA for anti-spike IgG antibodies. Sera were collected prior to the prime (pre), after prime (PB1), and after the boost (PB2). The S1 subunit of SARS-CoV-2 (involved in receptor mediated binding; Trenzyme SKU:P2020-022), the entire S2 subunit (R&D Systems, Cat# 10584-CV), or the trimer as Hexapro was used in homologous immunizations with a prime and a boost. Immunizations with the full trimer, the trimer subunit S1, or the trimer subunit S2 elicited anti-spike IgG antibodies, especially after the boost (PB2). Interestingly, mice primed and then boosted with the S2 subunit generated antibodies against the problematic autoantibody-inducing peptide (anti-B8 IgG). This was unique to the B8 peptide and strongly suggests that it is exposed as a linear peptide in the S2 subunit.

To test for the development of circulating IgG antibodies against the individual peptides or to the spike protein, we performed ELISAs against serum from animals in our immunization study. Pre-immunization (Pre) sera were compared against sera from primed mice (PB1), from primed and boosted mice (PB2), and from a second boost (PB3). Among the peptides that passed the immunogenicity prediction test, most generated corresponding anti-peptide IgGs in either homologous or heterologous immunization arms (data not shown), but only a subset generated anti-spike IgGs (shown in **Figure 1**). As expected, boosting enhanced anti-spike IgG responses in those eliciting initial antibody responses (**Figure 1**). Furthermore, a second boost significantly enhanced the response for several of the peptide-immunized groups (**Figure 1**). The peptides that elicited anti-spike IgGs were located in the spike protein as follows: (1) B10, B15, and B16, which are near the receptor binding domain (B15 and B16 are nearly identical except for a mutation found in later strains; **Supplementary Figure S2**); (2) B12, which is embedded in the fusion peptide domain; (3) B14 is in a novel S1 region located 50 bps upstream of the furin cleavage site (FCS); (4) B8 and B13, which are peptides that encompass different parts of the stem helix region leading to the HR2 (B8 from herein to be referred to as LHR2-B8); and (5) B6, which is 90 bps downstream of the FCS (**Supplementary Figure S2**; **Figure 1**). Homologous immunizations with peptides elicited lower levels of anti-spike IgG antibodies that improved greatly with a second boost while heterologous immunization (that involved priming with the trimer followed by one or two boosts with a specific peptide) yielded the highest levels of anti-spike IgG (**Figure 1**). Some peptides fared better than others in homologous immunizations (B14, B8, and B13 vs. B10, B6, B12, B15, and B16) while others required heterologous immunization with a trimer primer (**Figure 1**). All immunizations benefited from a second boost with the corresponding peptide (**Figure 1**; **Supplementary Table S1**). As expected, homologous immunizations with the full trimer generated robust anti-spike IgG titers, as did those with just the S1 or the S2 subunit (**Figure 2**). Surprisingly, immunization with the S2 subunit alone (unlike immunization with the S1 subunit) also elicited IgG antibodies to the LHR2-B8 peptide, albeit at relatively lower, but clearly detectable titers (**Figure 2**). These results suggest that LHR2-B8 is sufficiently exposed in S2 and is an important sub-epitope of the region, such that mice immunized with the S2 subunit can generate antibodies that recognize the peptide alone. These results also suggest that this region is exposed as a linear peptide and may represent one or more important epitopes (at least in the absence of RBD) that could potentially be targeted (by the appropriate vaccine regimen) for productive, neutralizing responses or, alternatively, may be a decoy region (i.e., irrespective of vaccine protocol optimization) that leads to non-neutralizing antibodies and potentially detracts from other regions.

Importantly, a separate study developed a peptide map of regions that elicited strong anti-spike IgG responses in both patients of COVID-19 and vaccine recipients (13) that matched many of the regions we identified. Regions encompassing peptides B5, B7, B8, B9, B12, B13, and B14 showed at least some positivity for anti-spike IgG in that study (**Supplementary Table S2**). Particularly strong were regions encompassing B8 and B13. This suggests that, as expected, these regions are also immunogenic in human hosts and highlights the importance of the B8/B13 region in potentially contributing to autoantibody formation.

### The region near the HR2 domain elicited high levels of autoreactive antibodies

**Figure 3:**
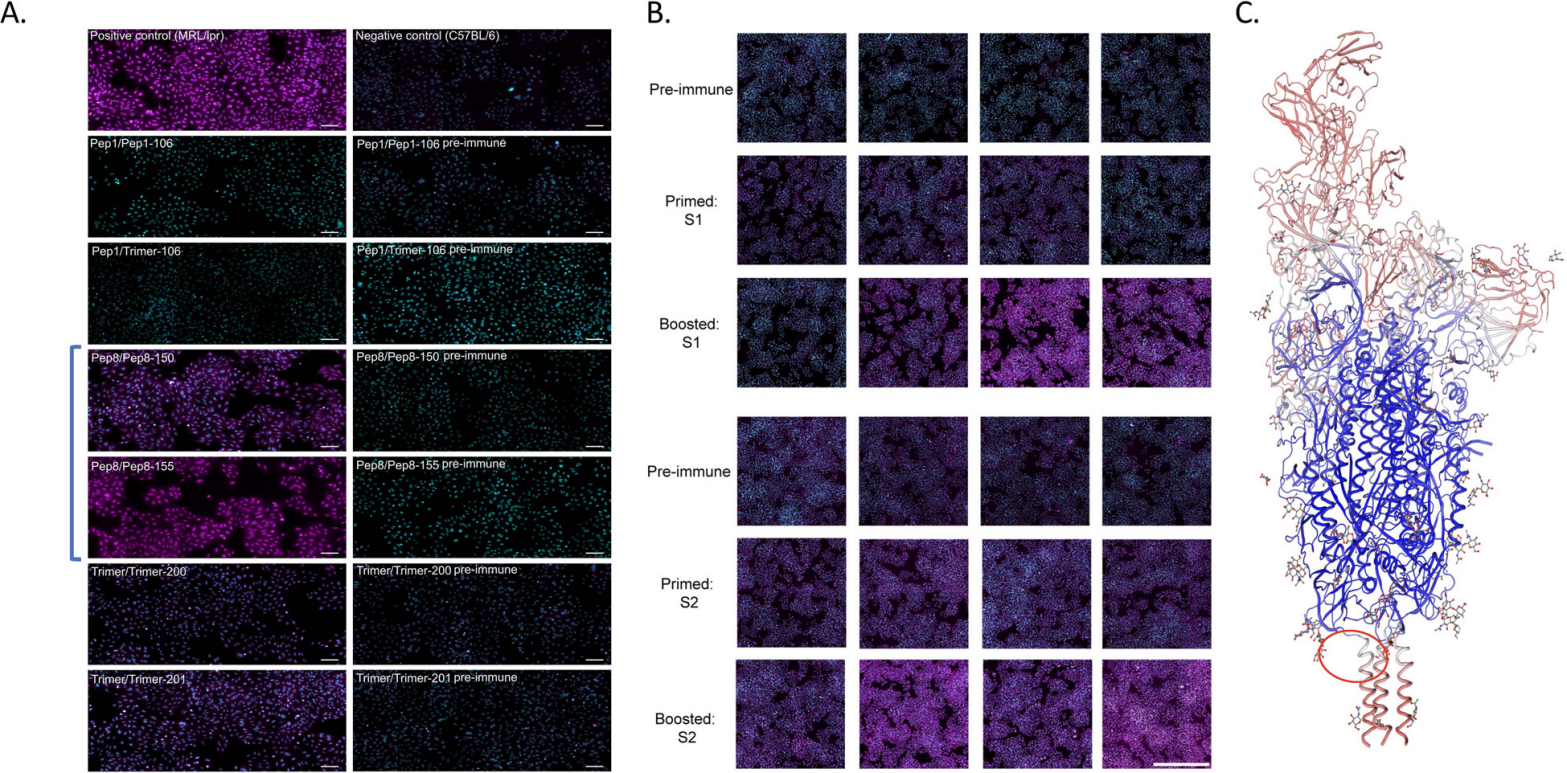
Antinuclear antigen (ANA) panels for mice injected with trimer or specific peptides. **(A)** Sera from mice immunized with most peptides were negative in an antinuclear autoantigen (ANA) assay when compared to pre-immunized sera. Each row depicts the results for immunization with either peptide twice (pep/pep), peptide and trimer (pep/trimer), or trimer twice (trimer/trimer), and this is reflected in the label for each panel followed by the mouse number. Mice immunized with the LHR2 (B8) peptide (samples Pep8/Pep8-150 and Pep8/Pep8-155; in brackets) were strongly positive for ANA. This was true for all mice receiving this peptide. Mouse 155, which died during the study (**Supplementary Figure S5**), had ANA positivity that was comparable to our positive control representing sera from MRL/lpr mice, a mouse model of SLE. All the other peptides were ANA negative (negative data only shown for peptide B1). The left panels are for sera from boosted mice with respective peptide or with trimer (P#2) while the right panels are for pre-immune sera (prior to any immunization including prime). Autoreactive antibodies are in magenta, while nuclei are seen in cyan, bar: 100 µm. Mice immunized with the Hexapro version of the spike protein trimer (trimer/trimer-200, trimer/trimer-201) also showed some autoreactivity albeit lower than mice immunized with LHR2-88 (Pep8 in figure). **(B)** ANA results for mice primed and boosted with the S1 subunit (top three panels) or the S2 subunit (bottom three panels). Each individual image reflects the results for a single mouse. Each row depicts the results for the same mouse serum collected 7 days after different points of immunization (pre-immune, prime, and boost) while each column depicts the results from different mice. The first two rows are males, while the last two are females. Autoreactive antibodies are in magenta, while nuclei are in cyan (bar: 1,000 µm). While both subunits showed positivity to ANA, mice immunized with the S2 subunit were slightly more positive, which correlated with the number of immunizations. **(C)** Spike model PODTC2 Swiss-Model Repository showing a region inducing autoantibodies (encircled in red) for one of the strands. This region is just upstream of the HR2 in the stem helix, and the autoantibody-inducing peptide in immunization was B8 (termed LHR2-B8).

Given the strong immunogenicity elicited by several of the peptide candidates chosen for testing in this study, we also tested sera from all groups for the presence of autoantibodies using the Hep2 antinuclear antigen test (ANA) adopted for mouse IgG antibodies as described previously (14). Results were compared against pre-immune sera. The majority of sera from homologous and heterologous immunized mice were largely negative in both tests (**Figure 3A**: 1-1-106 and 2-1-106 were representative of all negative samples) with a few sporadic exceptions in individual mice that revealed a typical distinct ring-like cytoplasmic pattern associated with the IMPDH2 gene in some cells, similar to that previously reported by ICAP (see ref. (10) and AC-23—Rods and Rings in https://www.anapatterns.org). However, serum from all mice immunized with peptides to the stem helix leading to HR2 (HR2-B8) was very strongly ANA positive (**Figure 3A**; **Table 1**). Immunization with the full trimer (as Hexapro) also elicited a positive ANA test that was not as strong as with the LHR2-B8 region-derived peptide alone (**Figure 3A**). Pre-immune sera were negative in all mice (**Figure 3A**), as was serum from KLH-only or adjuvant-only injected mice (**Supplementary Figure S3**). In addition, ANA positivity increased in intensity with each immunization step (**Supplementary Figure S3**). These results prove that B8 specifically elicited autoantibodies in these studies. In summary, peptides generated from the LHR2-B8 region consistently elicited high levels of autoreactive antibodies that were absent from pre-immune sera, while none of the other peptides elicited an overt autoreactive response. This peptide is in the stem helix directly N-terminal to the HR2 region (depicted in **Figure 3C**). The full trimer, the S1, and, more pronouncedly, the S2 subunits also elicited autoreactive antibodies, particularly after the boost (**Figure 3B**). While the autoreactivity generated by immunization with the S2 subunit may be explained by the presence of the LHR2-B8 region, it does not explain the autoreactivity-inducing activity of the S1 subunit. This suggests that an unidentified region (in our study) of the S1 subunit is also capable of eliciting some degree of autoreactive antibodies. Importantly however, neither of the whole spike proteins (Hexapro or vaccine prototype trimer) nor the subunits exhibited ANA positivity that was as intense and consistent as sera from mice immunized with the LHR2-B8 peptide.

**Table 1:** Autoantigen array results: sera from mice testing positive for ANA were examined for binding to common autoantigens associated with systemic autoimmune disorders and other diseases.

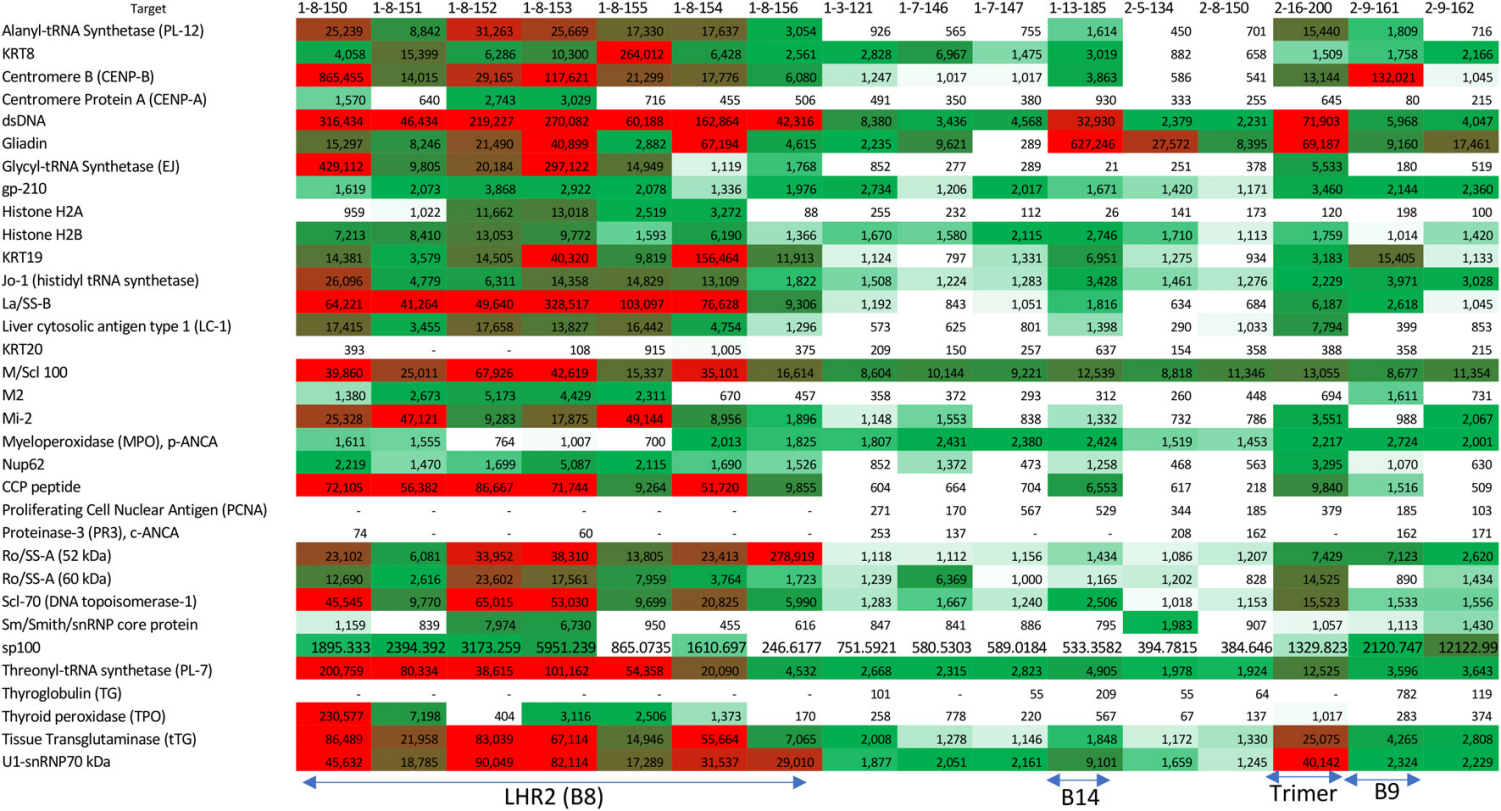

Shades of red imply strong binding. The color scale spans background binding levels (white, calculated as median + 3× standard deviation of negative control spots) through mild (light green) to intermediate (dark green) binding, to high binding levels (red, calculated as 90% of the maximal value). The mouse labels on the top correspond to whether they were immunized twice with peptide (#1) once with trimer then with peptide, or trimer twice (#2) followed by the peptide number and then the mouse number. Sera from all mice immunized with the LHR2-B8 peptide twice (1-8-150, 1-8-151, 1-8-152, 1-8-153, 1-8-155, 1-8-154, and 1-8-156) were strongly positive to multiple autoantigens. A mouse immunized with B8 once and then the trimer did not have any positive signals for self-antigen reactivity (2-8-150). Sera from a mouse immunized with B14, the full trimer, or B9 also showed some positivity to a few autoantigens. Most simple autoantigens in the panel, which are human derived, have high homology to mouse counterparts but a few do not, resulting in negative results for those inconclusive (**Supplementary Figure S4**). Complex autoantigens (protein/nucleic acid complexes for example) are of undetermined similarity but likely highly similar given the high degree of conservation of nucleic acids.

### Autoantibodies from mice immunized with peptides from the LHR2-B8 region-bound autoantigens detected in systemic autoimmune diseases like SLE and scleroderma

To identify which autoantigens may be associated with the positive ANA results, positive sera (even slightly so) were tested against an array of common autoantigens associated with autoimmunity. Sera from all mice immunized with LHR2-B8 peptide contained autoantibodies that recognized a variety of autoantigens associated with systemic autoimmunity in an autoarray assay (**Table 1**). Strikingly, all mice immunized with this peptide had high levels of anti-dsDNA autoantibodies, a hallmark of systemic lupus erythematosus (SLE) (15): They were also highly positive for La/SSB antigen (**Table 1**), a 47-kDa protein involved with small RNAs synthesized by RNA polymerase II, typically seen in Sjogren’s syndrome and SLE patients (16). Most sera from mice immunized with the LHR2-B8 peptide were also positive for an additional variety of disease associated autoantigens such as the PM/Scl100 antibodies seen in polymyositis and scleroderma patients [**Table 1**; (17, 18)]. Consistent with the ANA results, sera from mice immunized with other peptides were negative for most antigens. Also consistent with ANA results, some mice that received the Hexapro SARS2-CoV spike were positive for anti-dsDNA (SLE), U1-snRNP70 kDa [associated with SLE and mixed connective tissue disease (19)], gliadin [associated with celiac disease (20)], and, to a lesser degree, tissue transglutaminase [celiac disease (21)]. Sporadic positives were observed as follows (**Table 1**): (1) a single mouse that received a peptide containing part of the canonical HR2 region produced modest levels of anti-centromere B autoantibodies, typically seen in some scleroderma patients (22); (2) a single mouse in the group immunized with an S1 peptide upstream of the FCS (B14) showed low levels of anti-dsDNA and anti-gliadin antibodies; and (3) one of two mice immunized with the trimer and tested by ANA had significant autoantibodies to multiple autoantigens. Unique to the LHR2-B8 immunized mice, multiple autoantibodies were detected in all animals, suggesting that this is a potentially pathogenic region of the spike protein (**Figure 3A**; **Table 1**). Interestingly, peptide B13 is immediately adjacent to B8, yet did not elicit autoantibodies (data not shown). These results suggest that the specific amino acid composition of the B8 linear peptide is required for ANA positivity. Interestingly, two of seven mice that received homologous immunizations with the LHR2-B8 peptide died during the study. Necropsy of one of these mice revealed pathology consistent with an autoimmune etiology including vasculitis, skin tightening, and lesions, although autoimmunity could not be demonstrated as the cause of death (**Supplementary Figure S5**).

## Discussion

The ever-changing landscape of SARS-CoV-2 variants has necessitated a search for conserved epitopes capable of eliciting neutralizing antibodies to most if not all variants, as well as the design of immunogens that can specifically target them. Unfortunately, the goal of developing a safe pan-coronavirus vaccine may be problematic, as studies in other viruses have shown that some highly conserved regions are also prone to be autoreactive from molecular mimicry that could block the relevant B cells in development or, worse, contribute to autoimmune pathologies (2; 3). In this regard, data from patients with severe cases of SARS-CoV-2 infection revealed that many had developed autoantibodies to multiple autoantigens, suggesting that acquired autoimmunity may contribute to the severity of SARS-CoV-2 in some patients (23–26). To examine this issue in more detail, we systematically scanned the spike protein amino acid sequences for potential linear epitopes within regions of the spike protein that were highly conserved and tested them both for their immunogenicity and for their propensity to elicit autoantibodies, to inform pan-coronavirus vaccine efforts. We found that multiple epitopes across the S1 and S2 subunits of SARS-CoV-2 elicited potent anti-spike IgG antibody responses in simple two-step immunization schemes. These epitopes comprise five distinct regions in the spike, and most are highly conserved across coronaviruses, which is of obvious interest for efforts to develop vaccine protocols that can elicit antibody responses with broad cross-neutralizing activity. While these data are consistent with such peptides being capable of eliciting antibodies to the whole spike protein, it remains to be seen if conformational epitopes and/or multiple immunization steps may be needed for broad neutralization of SARS-CoV-2. In this regard, while we found that heterologous two-shot immunizations in which peptides were alternated with the full trimer work better than homologous immunization with peptides, three-shot homologous peptide immunizations elicited a response similar to, if not better than, the two-shot heterologous immunization schemes. Since a strong correlation has been previously found with anti-spike IgG titers and neutralization activity (27), it will be interesting to determine if multiple homologous immunizations using one or more of these peptide candidates are enough to eventually lead to neutralization of multiple SAS-CoV-2 variants or whether a conformational immunogen housing these linear epitopes (for example, in their native membrane context) is needed.

Most relevant to our study’s overall goal, however, is our finding that among all linear epitopes tested, the stem helix region adjacent to the HR2 induced very high levels of autoantibodies as determined by ANA and autoantigen arrays. Several patterns were evident from these mice: females were more positive by ANA than males, the positivity of the ANA increased with the number of immunizations, and every mouse immunized with this peptide had some degree of ANA positivity. The pattern was both cytoplasmic and nuclear, and the autoantigen array results revealed binding to multiple autoantigens with sera from these mice, particularly to autoantigens typically associated with connective tissue-associated autoimmune diseases such as SLE, Sjogren’s syndrome, and scleroderma. These include anti-dsDNA IgG, anti-La/SSB antigen IgG, and anti-PM/Scl100 IgG, among others. Immunization with the full trimer also elicited a positive ANA signal but one not as strong as in mice immunized with the LHR2-B8 peptide alone. In addition, immunization with the S2 subunit alone elicited a strong ANA signal that likely maps to the stem helix region housing the B8 peptide, the topic of ongoing studies. This notion is supported by the fact that mice immunized with the S2 subunit developed antibodies that recognized and bound the LHR2-B8 peptide by ELISA, suggesting that this region is exposed in S2 and is likely a dominant epitope, especially in the absence of the RBD. These results will have to be confirmed in human patients, but given the ancient nature of many of the self-antigens identified (such as dsDNA), this region is expected to elicit autoantibodies in human patients. While it is possible that nonspecific effects from conjugation with KLH or preparation of the peptide may contribute to this finding, we are confident that this is unlikely since the peptide was independently prepared, and we tested three separate times and none of the other similarly prepared peptides tested positive for widespread antinuclear antibodies through ANA testing. Indeed, neutralizing antibodies have been isolated from COVID-19 patients that generally map to this region (28).

While not all autoantibodies are pathogenic, data indicate that patients with severe SARS-CoV-2 infection as well as some patients with long Covid are highly positive in ANA assays. Two out seven mice receiving LHR2-B8 peptides in our studies died during our study with pathology consistent with inflammation, and skin changes associated with autoimmune disorders, albeit a causation link, was not established.

Combined, these data implicate the possibility that these autoreactivity-inducing regions may be associated with the acquired autoimmunity observed in some severe cases of SARS-CoV-2 infection [a phenomenon observed even at the earliest stages of the pandemic (29–32)], and especially if viral debris during infection exposes parts of S2 to the immune response. Indeed, various investigators have proposed that incomplete fractions of the spike protein may induce amyloidogenesis of neuro proteins or even induce excessive blood clotting (31, 32). Interestingly, a region similar to the stem helix/HR2 in HIV and in influenza has also been implicated in autoantibody generation (2, 3, 33–36). Since several studies have isolated neutralizing antibodies that bind epitopes to this region (13, 28), it is likely that this is a problematic region as it is both immunogenic and has high autoimmunogenic potential in humans. These results suggest that novel pan-coronavirus vaccine approaches, especially when seeking highly conserved epitopes outside the RBD, should either labor to avoid this region altogether or seek to mitigate (and/or “decouple”) its autoantibody-inducing effects from those that are potentially capable of broad neutralizing function, as we have previously described as theoretically feasible (i.e., “bnAb redemption”) for the HIV bnAb CH103 (37).

## Data Availability

The raw data supporting the conclusions of this article will be made available by the authors, without undue reservation.

## Supplementary material

Supplementary Table 1 ELISA results for individual mice treated with spike region peptides in homologous and heterologous immunizations (per **Figure 1**).

Supplementary Table 2 Mouse sera for Anti-Spike IgG ELISA results in this study compared to previous study with human samples. Keys: +++ 5-10 fold increase over pre-bleed; ++ 2-5 fold increase over pre-bleed; *after 2 boosts; #B1,B2, and B3 were redesigned as B15 and B16; ## data from Barber et al., 2022 [Ref# (13)].

Supplementary Figure 1 Immunization scheme for the generation of anti-peptide IgG antibodies in mice immunized with various peptides. **(A)** Predicted linear epitopes were used to design peptides (B1-B14) throughout the SARS-CoV-2 spike region conjugated to KLH. Peptides with predicted immunogenicity, were used to immunize mice and were examined for development of anti-peptide IgG in ELISA plates coated with corresponding peptide conjugated to BSA. Mice were either prime and boosted with the same peptide twice (homologous immunizations) or with hexapro followed by peptide (heterologous immunization). Pre-immunization sera (pre) was collected for all mice as well after the prime (Postbleed #1) and the boosts (Postbleed #2 and Postbleed#3). A third boost was done at day 138 post prime and sera collected 10 days later. **(B)** Control mice were immunized with either KLH only or Adjuvant only and tested for reactivity to any of the peptides. Control mice displayed no reactivity for any of the time points.

Supplementary Figure 2 Multiple alignment of the spike region of SARS-COV-2 identified multiple highly conserved regions. Selected linear epitopes are in italics and boldfaced. Some epitopes are overlapping. SARS-CoV-2 strains alignment used SARs T-Coffee: A novel method for multiple sequence alignments Notredame, Higgins, Heringa, JMB, 302 (205-217) 2000. The sequences for SARSCoV-2 and SARS spike were taken from UniProt P0DTC2 and P59594, respectively. BA.1 and BA.5 were taken from genbank sequences UFO69279.1 and UOZ45804.1.

Supplementary Figure 3 ANA test results (global view of wells) for mice immunized with LHR2-B8 peptide and KLH controls are displayed. Bound mouse IgG antibodies are in magenta. **(A)** Biding is seen in sera from mice primed and boosted with LHR2-B8 (B8). Negligible binding similar to matched pre-immune controls is seen with sera from mice primed and boosted with KLH. **(B)** Sequential immunization with peptide LRH2-B8 correlated with ANA positivity which was highest after the boost. Slide scanner images captured a portion of highly autofluorescent hydrophobic barrier (rim around wells). Bar = 1000 μm.

Supplementary Figure 4 Homology of array simple autoantigens (non-complexed proteins) between mouse and human are shown. Nucleic acids like dsDNA autoantigens are obviously the same in both species and among the most commonly seen in SLE, scleroderma and other rheumatic diseases.

Supplementary Figure 5 Mouse 155 received LHR2-B8 peptide both as prime and boost. Within days of the boost, it was found dead and necropsy revealed an autoimmune-like pathology. This mouse had ANA levels similar to MRL/lpr mice (**Figure 3A**) and similar skin lesions.
