# Supplementary material for: SARS-CoV-2 spike peptide analysis reveals a highly conserved region that elicits potentially pathogenic autoantibodies: implications to pan-coronavirus vaccine development
Source: Front Immunol. 2025 Feb 25;16:1488388. doi: 10.3389/fimmu.2025.1488388 (PMC11893414; doi:10.3389/fimmu.2025.1488388)
Supplement: Supplementary Table 1 — ELISA results for individual mice treated with spike region peptides in homologous and heterologous immunizations (per Figure 1 ). [file Table1.pdf]

PB2 ELISA FLuorescence

	Female	Female	Male	Male	Female	Female	Male	Female	Female	Male	Male	Female	Female	Male	Male
**Dilution**	MD503	MD504	MD505	MD506	MD507	MD508	MD509	MD511	MD512	MD513	MD514	MD515	MD516	MD517	MD518
**100**	9683.321	9349.944	9307.614	8591.927	9070.073	9225.612	9806.977	9235.34	9737.266	6204.52	8528.031	9170.488	9006.586	6906.482	7102.084
**400**	9932.422	9795.896	9098.925	7918.08	8210.716	9195.012	7713.576	8048.016	9228.226	4346.358	7208.28	8588.392	7839.465	6159.211	6555.379
**1600**	9803.537	9209.396	6085.374	4869.518	6155.357	7949.845	4641.719	5362.217	7241.881	2327.016	6063.072	6137.39	4650.55	5986.405	4732.992
**6400**	9060.505	8019.762	3768.017	2715.058	4222.263	5873.084	2792.36	4083.779	5302.703	1265.166	3440.622	4487.761	3657.586	3744.186	2695.405
**25600**	7469.63	6064.345	1777.614	1169.805	1834.058	3884.592	1499.49	2700.665	4322.144	635.604	2493.615	3088.927	2346.837	2103.26	1494.465
**102400**	6033.674	4727.69	1212.584	563.329	1122.256	2301.34	613.571	1480.215	2179.767	227.469	1419.776	1953.876	1429.299	1551.734	788.455

	Female	Female	Male	Female	Female	Male	Male	Female	Female	Male	Male	Female	Female	Male	Female	Female
**Dilution**	MD519	MD520	MD521	MD523	MD524	MD525	MD526	MD527	MD528	MD529	MD530	MD531	MD532	MD534	MD535	MD536
**100**	7746.141	8127.501	8568.628	7949.592	8400.585	1270.551	1450.682	1946.536	3058.946	1253.307	4959.918	1581.45	1529.454	7816.325	6777.841	3508.855
**400**	7293.727	7665.135	7574.644	7648.693	7653.64	698.764	871.867	1192.335	1947.68	844.857	3283.551	633.585	719.515	7088.258	5973.562	2176.999
**1600**	6074.216	7178.701	6650.323	6340.971	6435.279	418.623	577.213	791.154	1208.908	537.092	1977.485	431.543	565.07	6238.17	4609.676	840.406
**6400**	4296.562	5493.44	4728.238	4681.899	4854.565	154.757	179.733	287.272	389.918	165.053	1167.062	204.332	250.748	3196.126	1974.224	208.47
**25600**	2127.264	3335.837	2192.266	2709.331	2643.26	87.864	102.852	197.759	239.312	77.29	710.764	94.017	139.26	1974.162	840.834	83.799
**102400**	1081.401	2034.673	1081.257	1478.357	1507.951	17.859	26.598	64.153	124.957	44.657	262.824	48.435	65.278	868.723	482.745	40.857

PB3 ELISA FLuorescence

	Female	Female	Male	Male	Female	Female	Male	Female	Female	Male	Male	Female	Female	Male	Male
**Dilution**	MD503	MD504	MD505	MD506	MD507	MD508	MD510	MD511	MD512	MD513	MD514	MD515	MD516	MD517	MD518
**100**	15322.226	14908.085	14836.151	14902.569	14574.451	15272.167	14325.5	14056.058	13740.122	13577.185	10255.613	13615.824	13980.683	12920.656	13177.408
**400**	14931.523	13981.398	13561.634	13472.751	12767.86	14101.319	12731.57	12142.709	13170.306	12008.301	6941.678	12684.32	13539.732	12020.618	11774.59
**1600**	14381.443	12427.669	10437.377	9793.455	9312.91	11419.59	10231.83	10079.745	11303.674	9573.502	3698.424	10103.336	11650.614	8570.029	7815.807
**6400**	12397.946	9123.896	6693.245	5337.733	5146.5	6987.821	5844.697	6642.771	8302.368	5948.9	1241.74	6100.281	8498.169	6227.14	5306.011
**25600**	10348.255	5464.574	3483.774	2741.794	2292.532	2728.384	2453.585	3035.808	4630.999	2738.13	519.952	3048.022	4481.128	3208.208	2564.364
**102400**	6953.709	2791.377	1525.344	1261.485	993.163	1450.201	1437.402	1568.671	2236.369	1183.235	225.044	1357.618	2230.008	1322.509	1279.132

	Female	Female	Male	Male	Female	Female	Male	Male	Female	Female	Male	Male	Female	Female	Male	Female	Female
**Dilution**	MD519	MD520	MD521	MD522	MD523	MD524	MD525	MD526	MD527	MD528	MD529	MD530	MD531	MD532	MD534	MD535	MD536
**100**	12610.721	12995.752	1379.888	13443.97	13034.211	13125.653	11614.231	9461.64	12652.69	12083.299	8299.852	173.316	11714.61	9155.757	11779.16	12042.001	12182.215
**400**	11734.493	13322.027	493.877	13024.73	11993.148	12699.913	10587.932	6690.845	12222.81	11572.023	7458.371	95.917	10708.63	8023.085	11581.7	11343.845	11969.42
**1600**	8131.958	11958.947	58.8	10378.48	10234.629	9241.801	9127.239	3287.387	11209.19	11718.552	6396.05	51.458	9613.463	5232.31	9793.296	10599.188	10468.577
**6400**	4855.263	9886.394	31.365	7340.819	7615.062	5404.668	5534.408	1498.885	10048.5	10487.498	3985.525	28.414	3675.707	2455.781	7888.674	9792.754	8378.602
**25600**	2160.774	5972.196	20.871	4310.922	3741.53	2682.063	3036.645	634.153	7815.24	9293.142	2448.972	15.431	1805.413	1199.954	3415.545	7221.294	4172.935
**102400**	1152.172	3898.857	27.145	2022.071	2014.779	1279.916	1133.881	292.049	4664.65	6880.126	1118.86	27.057	647.78	343.263	1493.914	3848.779	1578.804

Peptide:	B6	B10	B12	B15	B16	B8	B13	B14
	MD505	MD509	MD513	MD517	MD521	MD525	MD529	MD534
	MD506	MD510	MD514	MD518	MD522	MD526	MD530	MD535
	MD507	MD511	MD515	MD519	MD523	MD527	MD531	MD536
	MD508	MD512	MD516	MD520	MD524	MD528	MD532	

Hexapro

MD503
MD504
