# Supplementary material for: SARS-CoV-2 spike peptide analysis reveals a highly conserved region that elicits potentially pathogenic autoantibodies: implications to pan-coronavirus vaccine development
Source: Front Immunol. 2025 Feb 25;16:1488388. doi: 10.3389/fimmu.2025.1488388 (PMC11893414; doi:10.3389/fimmu.2025.1488388)
Supplement: Supplementary Table 2 — Mouse sera for Anti-Spike IgG ELISA results in this study compared to previous study with human samples. Keys: +++ 5-10 fold increase over pre-bleed; ++ 2-5 fold increase over pre-bleed; *after 2 boosts; #B1,B2, and B3 were redesigned as B15 and B16; ## data from Barber et al., 2022 [Ref# (13)]. [file Table2.pdf]

Peptide name (sequence)	Location within spike sequence (subunit)	Anti-peptide-BSA (this study)	Anti-Spike IgG mouse (this study)##	Anti-Spike ab's (%human samples)	Region interval in Human study
SARS2- B5: ILPDPSKPSKRS	805(S2)	+++	+	yes (62%) (strong signal)	792-832
SARS2-B6: PLLTAEMIAQ	861 (S2)	+++	+++*	no	841-888
SARS2-B7: KIQDSLSSTASAL	933 (S2)	+++	+	yes (10%)	932-972
SARS2-B8: VYDPLQPELDSF	1136 (S2)	+++	+++*	yes (94%) (strong signal)	1128-1168
SARRS2-B9: KNHTSPDVDLGD	1156 (S2)	+++	+	yes (50%)	1156-1196
SARS2-B10: AWNSNNLDSKV	433 (S1)	+++	+++*	no	428-468
SARS2-B12 : IEDLLFNKVTLAD	804 (S2)	+++	+++*	yes (56%) (strong signal)	792-832
SARS2-B13 : KEELDKYFKN	1148 (S2)	+++	+++*	yes (94%) (strong signal)	1128-1168
SARS2-B14: LYQDVNCTEVPVAIHADQLTPTWRVY	611 (S1)	+++	+++*	yes (37.5) (moderate signal)	596-636
SARS-B15: RGDEVRQIAPGQTGKIADYNYKLPDC	401 (S1)	+++	+++*	no	400-440
SARS-B16: RGDEVSQIAPGQTGNIADYNYKLPD (same as B15 with variant)	401 (S1)	+++	+++*	no	400-440

+++ 5-10 fold increase over pre-bleed

++ 2-5 fold increase over pre-bleed

* after 2 boosts

#B1,B2, and B3 : were redesigned as B15 and B16

data from Barber et al., 2022 (Ref#13)
