# Supplementary material for: SARS-CoV-2 spike peptide analysis reveals a highly conserved region that elicits potentially pathogenic autoantibodies: implications to pan-coronavirus vaccine development
Source: Front Immunol. 2025 Feb 25;16:1488388. doi: 10.3389/fimmu.2025.1488388 (PMC11893414; doi:10.3389/fimmu.2025.1488388)
Supplement: Supplementary Figure 1 — Immunization scheme for the generation of anti-peptide IgG antibodies in mice immunized with various peptides. (A) Predicted linear epitopes were used to design peptides (B1-B14) throughout the SARS-CoV-2 spike region conjugated to KLH. Peptides with predicted immunogenicity, were used to immunize mice and were examined for development of anti-peptide IgG in ELISA plates coated with corresponding peptide conjugated to BSA. Mice were either prime and boosted with the same peptide twice (homologous immunizations) or with hexapro followed by peptide (heterologous immunization). Pre-immunization sera (pre) was collected for all mice as well after the prime (Postbleed #1) and the boosts (Postbleed #2 and Postbleed#3). A third boost was done at day 138 post prime and sera collected 10 days later. (B) Control mice were immunized with either KLH only or Adjuvant only and tested for reactivity to any of the peptides. Control mice displayed no reactivity for any of the time points. [file SupplementaryFile1.pdf]

A.

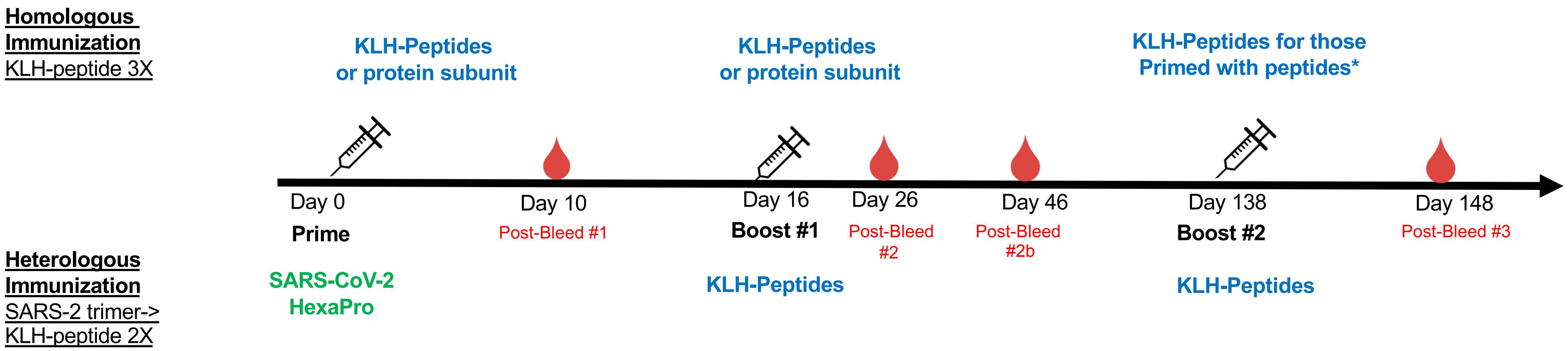

* Mice primed and boosted with protein subunits were only boosted once

B.

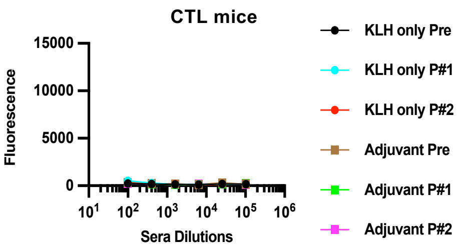

Mice primed and boosted with only KLH or adjuvant
failed to elicit anti-spike IgG antibodies

Supplemental Figure 1

```
                                                                                 B15, B16.      B10
SARS-CoV-2    RKRISNCVADYSVLYNSASFSTFKCYGVSPTKLNDLCFTNVYADSFVIRGDEVRQIAPGQTGKIADYNYKLPDDFTGCVIAWNSNNLDSK
B.1.1.529     RKRISNCVADYSVLYNLAPFFTFKCYGVSPTKLNDLCFTNVYADSFVIRGDEVRQIAPGQTGNIADYNYKLPDDFTGCVIAWNSNKLDSK
BA.5          RKRISNCVADYSVLYNFAPFFAFKCYGVSPTKLNDLCFTNVYADSFVIRGNEVSQIAPGQTGNIADYNYKLPDDFTGCVIAWNSNKLDSK
SARS          RKKISNCVADYSVLYNSTFFSTFKCYGVSATKLNDLCFSNVYADSFVVKGDDVRQIAPGQTGVIADYNYKLPDDFMGCVLAWNTRNIDAT
cons          **:************* : * :*******.********:********::*::* ******** ************ ***:***:.::*:.

SARS-CoV-2    VGGNYNYLYRLFRKSNLKPFERDISTEIYQAGSTPCNGVEGFNCYFPLQSYGFQPTNGVGYQPYRVVVLSFELLHAPATVCGPKKSTNLV
B.1.1.529     VSGNYNYLYRLFRKSNLKPFERDISTEIYQAGNKPCNGVAGFNCYFPLRSYSFRPTYGVGHQPYRVVVLSFELLHAPATVCGPKKSTNLV
BA.5          VGGNYNYRYRLFRKSNLKPFERDISTEIYQAGNKPCNGVAGVNCYFPLQSYGFRPTYGVGHQPYRVVVLSFELLHAPATVCGPKKSTNLV
SARS          STGNYNYKYRYLRHGKLRPFERDISNVPFSPDGKPCTPP-ALNCYWPLNDYGFYTTTGIGYQPYRVVVLSFELLNAPATVCGPKLSTDLI
cons            ***** ** :*:.:*:*******.  :.....**.   ..***:**..*.* .* *:*:*************:********* **:*:

                                                                                  B14
SARS-CoV-2    KNKCVNFNFNGLTGTGVLTESNKKFLPFQQFGRDIADTTDAVRDPQTLEILDITPCSFGGVSVITPGTNTSNQVAVLYQDVNCTEVPVAI
B.1.1.529     KNKCVNFNFNGLKGTGVLTESNKKFLPFQQFGRDIADTTDAVRDPQTLEILDITPCSFGGVSVITPGTNTSNQVAVLYQGVNCTEVPVAI
BA.5          KNKCVNFNFNGLTGTGVLTESNKKFLPFQQFGRDIADTTDAVRDPQTLEILDITPCSFGGVSVITPGTNTSNQVAVLYQGVNCTEVPVAI
SARS          KNQCVNFNFNGLTGTGVLTPSSKRFQPFQQFGRDVSDFTDSVRDPKTSEILDISPCSFGGVSVITPGTNASSEVAVLYQDVNCTDVSTAI
cons          **:*********.****** *.*:* ********::* **:****:* *****:***************:*.:******.****:*..**

SARS-CoV-2    HADQLTPTWRVYSTGSNVFQTRAGCLIGAEHVNNSYECDIPIGAGICASYQTQTNSPRRARSVASQSIIAYTMSLGAENSVAYSNNSIAI
B.1.1.529     HADQLTPTWRVYSTGSNVFQTRAGCLIGAEYVNNSYECDIPIGAGICASYQTQTKSHRRARSVASQSIIAYTMSLGAENSVAYSNNSIAI
BA.5          HADQLTPTWRVYSTGSNVFQTRAGCLIGAEYVNNSYECDIPIGAGICASYQTQTKSHRRARSVASQSIIAYTMSLGAENSVAYSNNSIAI
SARS          HADQLTPAWRIYSTGNNVFQTQAGCLIGAEHVDTSYECDIPIGAICASYHTVS----LLRSTSQKSIVAYTMSLGADSSIAYSNNTIAI
cons          *******:**:****.*****:********:*:.****************:* :      **.:.:**:********:.*:*****:***

SARS-CoV-2    PTNFTISVTTEILPVSMTKTSVDCTMYICGDSTECSNLLLQYGSFCTQLNRALTGIAVEQDKNTQEVFAQVKQIYKTPPIKDFGGFNFSQ
B.1.1.529     PTNFTISVTTEILPVSMTKTSVDCTMYICGDSTECSNLLLQYGSFCTQLKRALTGIAVEQDKNTQEVFAQVKQIYKTPPIKYFGGFNFSQ
BA.5          PTNFTISVTTEILPVSMTKTSVDCTMYICGDSTECSNLLLQYGSFCTQLKRALTGIAVEQDKNTQEVFAQVKQIYKTPPIKYFGGFNFSQ
SARS          PTNFSISITTEVMPVSMAKTSVDCNMYICGDSTECANLLLQYGSFCTQLNRALSGIAAEQDRNTREVFAQVKQMYKTPTLKYFGGFNFSQ
cons          ****:**:***::****:******.**********:*************:***:***.***:**:********:****.:* ********

                  B5, B12                                                 B6
SARS-CoV-2    ILPDPSKPSKRSFIEDLLFNKVTLADAGFIKQYGDCLGDIAARDLICAQKFNGLTVLPPLLTDEMIAQYTSALLAGTITSGWTFGAGAAL
B.1.1.529     ILPDPSKPSKRSFIEDLLFNKVTLADAGFIKQYGDCLGDIAARDLICAQKFKGLTVLPPLLTDEMIAQYTSALLAGTITSGWTFGAGAAL
BA.5          ILPDPSKPSKRSFIEDLLFNKVTLADAGFIKQYGDCLGDIAARDLICAQKFNGLTVLPPLLTDEMIAQYTSALLAGTITSGWTFGAGAAL
SARS          ILPDPLKPTKRSFIEDLLFNKVTLADAGFMKQYGECLGDINARDLICAQKFNGLTVLPPLLTDDMIAAYTAALVSGTATAGWTFGAGAAL
cons          ***** **:********************:****:***** **********:***********:*** **:**::** *:**********

SARS-CoV-2    QIPFAMQMAYRFNGIGVTQNVLYENQKLIANQFNSAIGKIQDSLSSTASALGKLQDVVNQNAQALNTLVKQLSSNFGAISSVLNDILSRL
B.1.1.529     QIPFAMQMAYRFNGIGVTQNVLYENQKLIANQFNSAIGKIQDSLSSTASALGKLQDVVNHNAQALNTLVKQLSSKFGAISSVLNDIFSRL
BA.5          QIPFAMQMAYRFNGIGVTQNVLYENQKLIANQFNSAIGKIQDSLSSTASALGKLQDVVNHNAQALNTLVKQLSSKFGAISSVLNDILSRL
SARS          QIPFAMQMAYRFNGIGVTQNVLYENQKQIANQFNKAISQIQESLTTTSTALGKLQDVVNQNAQALNTLVKQLSSNFGAISSVLNDILSRL
cons          *************************** ******.**.:**:**::*::**********:*************:***********:***

SARS-CoV-2    DKVEAEVQIDRLITGRLQSLQTYVTQQLIRAAEIRASANLAATKMSECVLGQSKRVDFCGKGYHLMSFPQSAPHGVVFLHVTYVPAQEKN
B.1.1.529     DKVEAEVQIDRLITGRLQSLQTYVTQQLIRAAEIRASANLAATKMSECVLGQSKRVDFCGKGYHLMSFPQSAPHGVVFLHVTYVPAQEKN
BA.5          DKVEAEVQIDRLITGRLQSLQTYVTQQLIRAAEIRASANLAATKMSECVLGQSKRVDFCGKGYHLMSFPQSAPHGVVFLHVTYVPAQEKN
SARS          DKVEAEVQIDRLITGRLQSLQTYVTQQLIRAAEIRASANLAATKMSECVLGQSKRVDFCGKGYHLMSFPQAAPHGVVFLHVTYVPSQERN
cons          **********************************************************************:**************:**:*

                                                                                     B8, B13
SARS-CoV-2    FTTAPAICHDGKAHFPREGVFVSNGTHWFVTQRNFYEPQIITTDNTFVSGNCDVVIGIVNNTVYDPLQPELDSFKEELDKYFKNHTSPDV
B.1.1.529     FTTAPAICHDGKAHFPREGVFVSNGTHWFVTQRNFYEPQIITTDNTFVSGNCDVVIGIVNNTVYDPLQPELDSFKEELDKYFKNHTSPDV
BA.5          FTTAPAICHDGKAHFPREGVFVSNGTHWFVTQRNFYEPQIITTDNTFVSGNCDVVIGIVNNTVYDPLQPELDSFKEELDKYFKNHTSPDV
SARS          FTTAPAICHEGKAYFPREGVFVFNGTSWFITQRNFFSPQIITTDNTFVSGNCDVVIGIINNTVYDPLQPELDSFKEELDKYFKNHTSPDV
cons          *********:***:******** *** **:*****:.*********************:*******************************

SARS-CoV-2    DLGDISGINASVVNIQKEIDRLNEVAKNLNESLIDLQELGKYEQYIKWPWYIWLGFIAGLIAIVMVTIMLCCMTSCCSCLKGCCSCGSCC
B.1.1.529     DLGDISGINASVVNIQKEIDRLNEVAKNLNESLIDLQELGKYEQYIKWPWYIWLGFIAGLIAIVMVTIMLCCMTSCCSCLKGCCSCGSCC
BA.5          DLGDISGINASVVNIQKEIDRLNEVAKNLNESLIDLQELGKYEQYIKWPWYIWLGFIAGLIAIVMVTIMLCCMTSCCSCLKGCCSCGSCC
SARS          DLGDISGINASVVNIQKEIDRLNEVAKNLNESLIDLQELGKYEQYIKWPWYVWLGFIAGLIAIVMVTILLCCMTSCCSCLKGACSCGSCC
```

Supplemental Figure 2

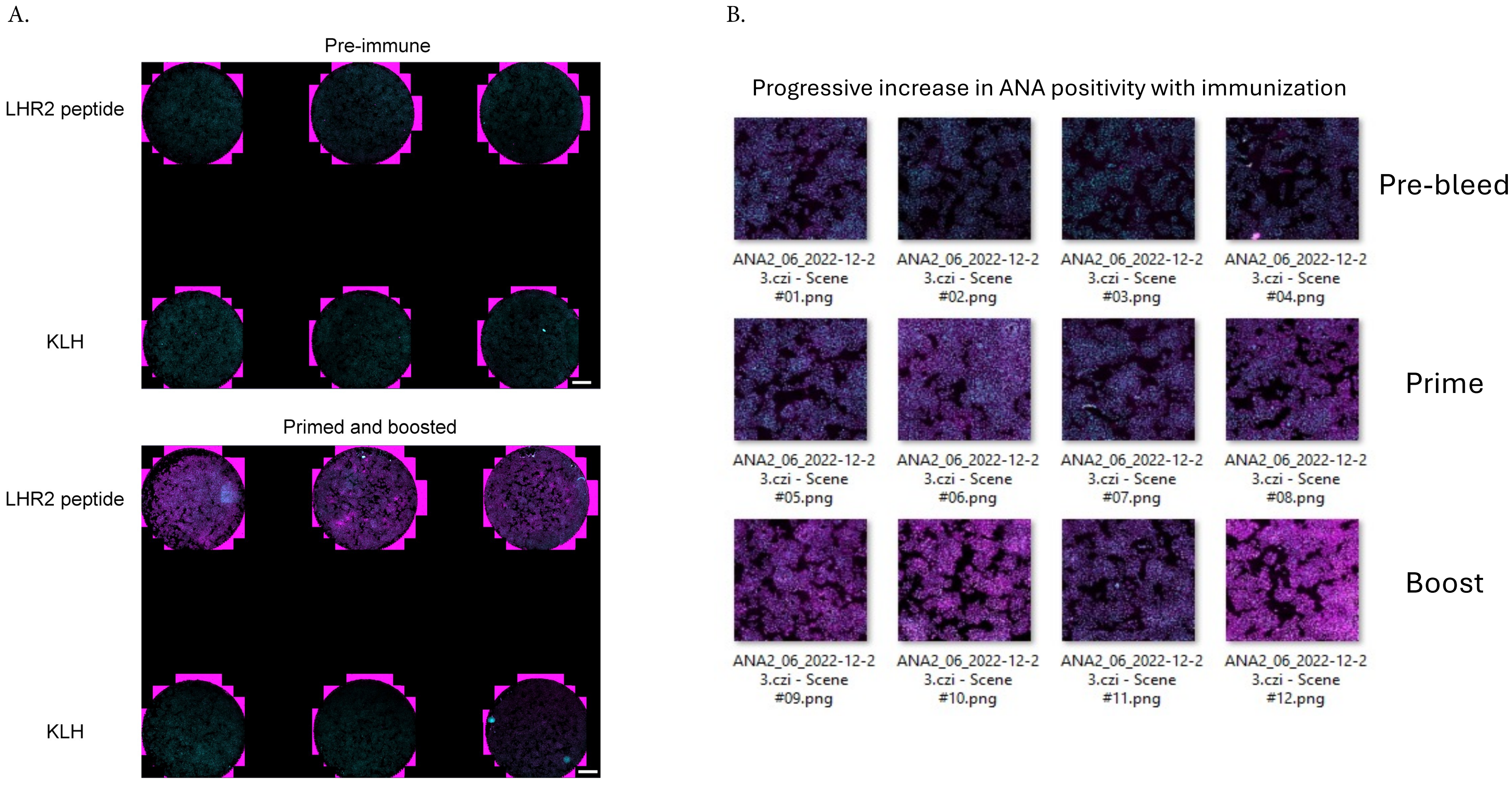

A.
Pre-immune
LHR2 peptide
KLH
Primed and boosted
LHR2 peptide
KLH
B.
Progressive increase in ANA positivity with immunization
ANA2_06_2022-12-23.czi - Scene #01.png
ANA2_06_2022-12-23.czi - Scene #02.png
ANA2_06_2022-12-23.czi - Scene #03.png
ANA2_06_2022-12-23.czi - Scene #04.png
Pre-bleed
ANA2_06_2022-12-23.czi - Scene #05.png
ANA2_06_2022-12-23.czi - Scene #06.png
ANA2_06_2022-12-23.czi - Scene #07.png
ANA2_06_2022-12-23.czi - Scene #08.png
Prime
ANA2_06_2022-12-23.czi - Scene #09.png
ANA2_06_2022-12-23.czi - Scene #10.png
ANA2_06_2022-12-23.czi - Scene #11.png
ANA2_06_2022-12-23.czi - Scene #12.png
Boost

Gene name	Human to mouse Uniprot ALIGN tool percent identity matrix	**Mapped array protein**		
CALCA	80.16			
CENPA	71.64	Centromere Protein A (CENP-A)		
CENPB	92.45	Centromere B (CENP-B)		
GARS	92.32	Glycyl-tRNA Synthetase (EJ)		**Uncertain array antigens**
HARS	94.89	Jo-1 (histidyl tRNA synthetase)		Alanyl-tRNA Synthetase (PL-12)
KRT19	82.75	KRT19		Gliadin
KRT20	79.72	KRT20		gp-210
KRT8	90.64	KRT8		Histone H2A
MPO	86.35	Myeloperoxidase (MPO), p-ANCA		Histone H2B
NUP210	84.51			Liver cytosolic antigen type 1 (LC-1)
NUP62	80.27	Nup62		M/Scl 100
PCNA	96.93	Proliferating Cell Nuclear Antigen (PCNA)		M2
PRTN3	65.08	Proteinase-3 (PR3), c-ANCA		Mi-2
SNRNP70	97.93			CCP peptide
SP100	40.38	sp100		Ro/SS-A (52 kDa)
SSB	81.7	La/SS-B		Ro/SS-A (60 kDa)
TARS2	84.26			Sm/Smith/snRNP core protein
TG	74.28	Thyroglobulin (TG)		Threonyl-tRNA synthetase (PL-7)
TGM2	84.26	Tissue Transglutaminase (tTG)		Thyroid peroxidase (TPO)
THPO	71.39			U1-snRNP70 kDa
TOP1	99.22	Scl-70 (DNA topoisomerase-1)		
TRIM21	69.53			

Supplemental Figure 4

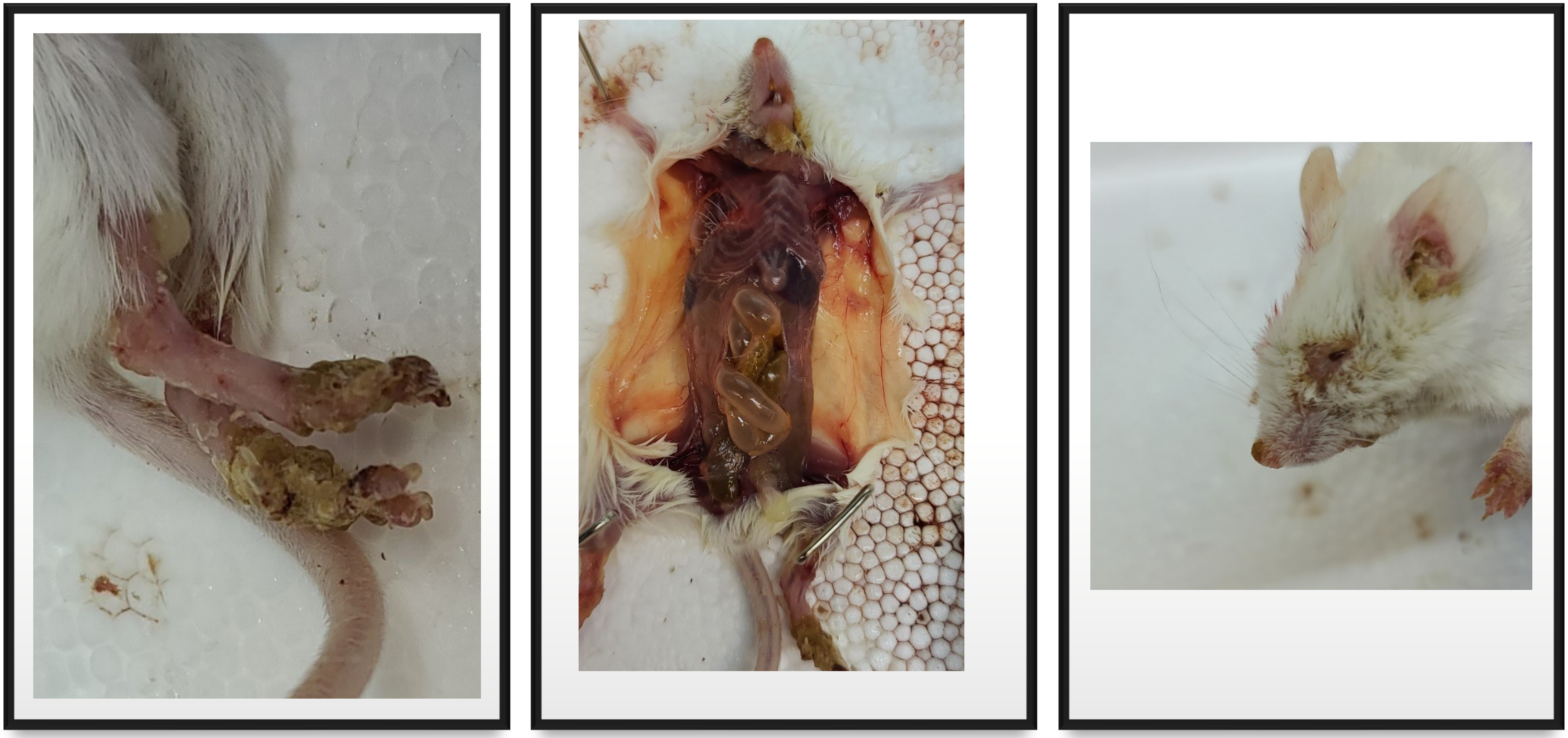

Supplemental Figure 5
